# Connectivity Map Analysis of a Single-Cell RNA-Sequencing -Derived Transcriptional Signature of mTOR Signaling

**DOI:** 10.3390/ijms22094371

**Published:** 2021-04-22

**Authors:** Naim Al Mahi, Erik Y. Zhang, Susan Sherman, Jane J. Yu, Mario Medvedovic

**Affiliations:** 1 Division of Biostatistics and Bioinformatics, Department of Environmental and Public Health Sciences, University of Cincinnati College of Medicine, Cincinnati, OH 45267, USA; mahina@mail.uc.edu; 2 AbbVie Inc., North Chicago, IL 60064, USA; 3 Division of Pulmonary, Critical Care and Sleep Medicine, University of Cincinnati College of Medicine, Cincinnati, OH 45267, USA; zhangey@ucmail.uc.edu (E.Y.Z.); yuj9@ucmail.uc.edu (J.J.Y.); 4 The LAM Foundation, Cincinnati, OH 45242, USA; ssherman@thelamfoundation.org; 5 Department of Biomedical Informatics, University of Cincinnati College of Medicine, Cincinnati, OH 45267, USA

**Keywords:** lymphangioleiomyomatosis, single-cell, LINCS, connectivity analysis, mTOR

## Abstract

In the connectivity map (CMap) approach to drug repositioning and development, transcriptional signature of disease is constructed by differential gene expression analysis between the diseased tissue or cells and the control. The negative correlation between the transcriptional disease signature and the transcriptional signature of the drug, or a bioactive compound, is assumed to indicate its ability to “reverse” the disease process. A major limitation of traditional CMaP analysis is the use of signatures derived from bulk disease tissues. Since the key driver pathways are most likely dysregulated in only a subset of cells, the “averaged” transcriptional signatures resulting from bulk analysis lack the resolution to effectively identify effective therapeutic agents. The use of single-cell RNA-seq (scRNA-seq) transcriptomic assay facilitates construction of disease signatures that are specific to individual cell types, but methods for using scRNA-seq data in the context of CMaP analysis are lacking. Lymphangioleiomyomatosis (LAM) mutations in TSC1 or TSC2 genes result in the activation of the mTOR complex 1 (mTORC1). The mTORC1 inhibitor Sirolimus is the only FDA-approved drug to treat LAM. Novel therapies for LAM are urgently needed as the disease recurs with discontinuation of the treatment and some patients are insensitive to the drug. We developed methods for constructing disease transcriptional signatures and CMaP analysis using scRNA-seq profiling and applied them in the analysis of scRNA-seq data of lung tissue from naïve and sirolimus-treated LAM patients. New methods successfully implicated mTORC1 inhibitors, including Sirolimus, as capable of reverting the LAM transcriptional signatures. The CMaP analysis mimicking standard bulk-tissue approach failed to detect any connection between the LAM signature and mTORC1 signaling. This indicates that the precise signature derived from scRNA-seq data using our methods is the crucial difference between the success and the failure to identify effective therapeutic treatments in CMaP analysis.

## 1. Introduction

Currently, mTORC1 inhibitor sirolimus is the only drug approved by the Food and Drug Administration (FDA) that improves pulmonary dysfunction and decelerates LAM progression in most patients [1]. However, sirolimus treatment does not lead to progression-free survival and has a cytostatic rather than a cytocidal effect. Lung function decline resumes following drug discontinuation and thus uninterrupted drug exposure is required for prolonged benefit [1,2]. The drug cannot completely eliminate LAM cells, potentially because chronic exposure to sirolimus induces refractoriness and resistant behavior of mTORC1-hyperactive LAM cells [3]. Therefore, it is urgent to identify remission-inducing and durably effective therapeutic agents for LAM.

As an alternative to de novo drug discovery, identifying new therapeutic uses of existing drugs by leveraging large compendia of biomedical data, also known as drug repositioning, has been used as a potential tool in drug discovery and development [4,5,6]. In the connectivity map (CMap) drug repositioning [7], the transcriptional signature of disease is constructed by differential gene expression analysis between the diseased tissue or cells and the control. The negative correlation between the transcriptional disease signature and the transcriptional signature of the drug treatment is used to identify drugs capable of “reversing” the disease process to be used as potential therapeutics. For example, histone deacetylase (HDAC) inhibitor vorinostat, which is known to treat cutaneous T-cell lymphoma, has been shown to be effective in treating gastric cancer [8], and drug topiramate has been identified as a potential candidate to treat inflammatory bowel disease (IBD) by comparing gene expression signatures of IBD against drug perturbational signatures [9]. The most recent edition of the connectivity map library, generated by the integrated network-based cellular signatures (LINCS) project, catalogues transcriptional signatures of more than 20,000 drugs and uncharacterized small chemicals across 77 cell lines facilitating drug repositioning and identification of new therapeutic agents [10,11].

A major limitation of traditional CMaP analysis is the use of signatures derived from bulk disease tissues. Since the key driver pathways are most likely dysregulated in only a subset of cells, the “averaged” transcriptional signatures resulting from bulk analysis lacks the resolution to effectively “connect” disease with effective therapeutic agents. With the recent progress of next-generation sequencing technologies, single-cell RNA-seq (scRNA-seq) has emerged as a powerful tool to investigate inter-cellular heterogeneity at single-cell level. The gene expression dynamics of individual cells provides means to study complex disease mechanisms at an unprecedented resolution. The use of single-cell RNA-seq (scRNA-seq) transcriptomic assay facilitates construction of disease signatures that are specific to individual cell types. Although considerable research has been devoted to using bulk transcriptional signatures for computational drug repositioning and methods for analysis of scRNA-seq data are being developed at breath-taking speed, methodologies for connecting diseases, genes, and drugs using scRNA-seq data are lacking.

In this paper, we present the complete protocol for performing connectivity analysis using scRNA-seq data, including signatures construction and connectivity analysis with individual drug signatures as well as the whole classes of drugs with the same mechanism of action. The methods are described in the context of CMaP of LAM scRNA-seq signatures. Our analyses successfully predict therapeutic effects of currently used drugs inhibiting mTORC1 signaling. Importantly, we demonstrate that these results are contingent on use of scRNA-seq data and our methods for constructing single-cell disease signature and would not be possible by connectivity analysis of standard bulk RNA-seq disease signatures.

## 2. Results

### 2.1. Overview of scRNA-Seq Connectivity Analysis

Conventional transcriptome profiling methods such as bulk RNA-seq rely on averaging molecular signals across a large population of cells. The goal of our analysis methods is to construct a transcriptional signature of disease-critical cells, which may represent only a small fraction of profiled cells, by comparing them to the matched cell type in the control non-diseased tissue. Such a disease signature factors out the cell-type to cell-type variability and facilitates identification of effective therapeutics when the standard connectivity analysis of bulk disease signatures fails.

The analytical workflow of scRNA-seq signature construction and connectivity analysis proceeds as (1) cluster analysis of disease and controls samples; (2) construction of cluster annotating signature (CAS) for each cluster in the disease sample and identification of the disease-critical cell subpopulation using the panel of disease marker genes; (3) identification of matching control cell populations in the non-diseased sample; (4) construction of disease-characterizing signature (DCS) by comparing the disease-critical cells with the matched control cells; and (5) “connecting” DCS to LINCS-L1000 chemical perturbational signatures. Here, we illustrate the methodology in the analysis in the context of the LAM scRNA-seq data. Technical details of each step are provided in the Methods and outlined in Supplementary Figure S1.

### 2.2. Identification of Distinct Cell Populations

scRNA-seq data were generated using 10× Chromium platform on dissociated lungs from one naïve LAM patient (LAM1), one sirolimus treated LAM patient (LAM2), and one normal patient (WT), respectively, and have been previously described and analyzed [12]. In total, 19,384 cells (7244 cells from LAM1, 6545 cells from LAM2, and 5595 cells from WT) passed quality control filters, with an average number of detected genes (UMI > 0) of 2089, 2466, and 1564 per cell in LAM1, LAM2, and WT, respectively (Supplementary Figure S2). The analytical workflow outlined above was carried out for LAM1 and LAM2 samples separately. Cluster analysis identified 19 clusters in each of the samples.

### 2.3. Construction of Cluster Annotating Signatures

**Figure 1:**
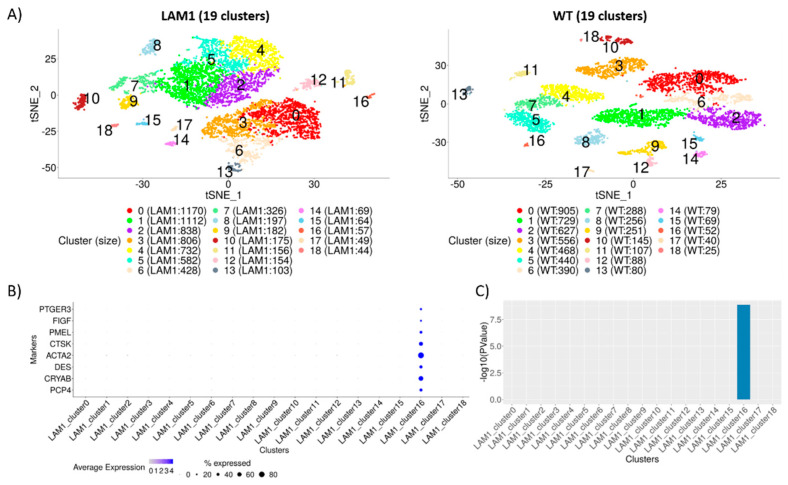
Cluster analysis of scRNA-seq samples. (**A**) Unsupervised clustering of 7244 cells from LAM1 (top panel) and 5595 cells from wild-type (WT) sample (bottom panel) is represented in two-dimensional t-SNE plots with perplexity 30. A total of 19 clusters were identified in each sample using Seurat’s graph-based clustering initialized with top principal components with largest variances. Clusters are colored and labeled distinctively, and the number of cells in each cluster is noted inside the parenthesis in the legends. (**B**) Expression of known LAM markers was used to identify the cluster of LAM cells, with the size of the dot representing the percentage of cells expressed, and color is proportional to the average expression of the genes. All the 8 markers show moderate to high expression in at least 30% cells in cluster 16 of LAM1. (**C**) Marker enrichment was conducted using Fisher’s exact test based on the significantly (FDR < 0.05) differentially expressed genes from each of the cluster annotating signatures of LAM1. All the markers were significantly DE only in cluster 16, whereas none of the markers were significant in any other cluster.

To construct cluster annotating signatures (CAS), pairwise comparisons for each cluster (Figure 1A) were conducted and then combined into a single cluster-specific signature (Methods; Supplementary Figure S1). The top most significantly (FDR < 0.05) up-regulated genes, namely, cluster annotating signature (CAS), were then used to annotate cell clusters. This step was iterated for each cluster separately, and lists of all cluster annotating genes are provided in Supplemental Tables.

To identify disease-critical cell sub-population, we utilized a set of eight marker genes identified as the markers of LAM from the literature (Figure 1B); Supplementary Table S1). All the markers were exclusively highly expressed in cluster 16 of LAM1 (Figure 1B), and this cluster was the only one whose signature was enriched for expression of the marker genes (Figure 1C; Supplementary Table S2), indicating that the cluster (herein denoted as LAM1_cluster16_) consists of LAM cells.

To further characterize cells in different clusters, we performed enrichment analysis [13] of the top 200 most significantly up-regulated genes from each cluster for cell type marker from three databases: human cell landscape (HCL) [14], cellMarker (CM) [15], and PanglaoDB (PDB) [16], and the tissue markers derived from the gene atlas dataset [17]. Top three most significantly (FDR < 0.05) enriched tissue and cell-type categories with log odds ratio above 1.5 from each cluster were selected for each cluster. Associations between the clusters and cell and tissue type are summarized in the Supplementary Figure S3. The analysis identified clusters of epithelial, endothelial, and immune cells. The LAM1_cluster16_ cells were also enriched for markers of mesenchymal and uterus cells signatures (Supplementary Figure S3). The list of all enriched pathways is provided in the Supplemental Tables.

### 2.4. Construction of Disease Characterizing Signature

**Figure 2:**
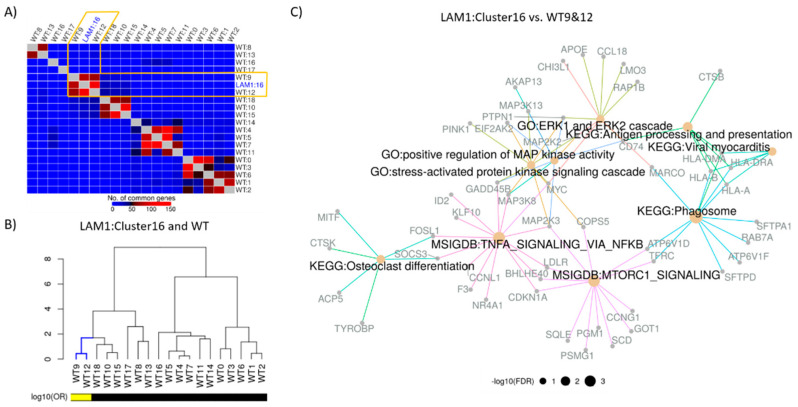
Construction and functional enrichment of disease characterizing signature. (**A**) WT clusters were matched with the LAM cluster in terms of top 200 most significantly (FDR < 0.05) up-regulated genes from each of the CAS. Unsupervised hierarchical clustering revealed sub-clusters of LAM and WT clusters, where LAM1_cluster16_ was clustered with WT clusters 9 and 12. (**B**) Significance of the overlaps between LAM and WT cell clusters based on the significantly (FDR < 0.05) up-regulated genes were assessed via Fisher’s exact test. Cluster similarities were measured using log10 odds ratio and hierarchical clustering of LAM1_cluster16_ vs. WT is visualized via dendrograms. Log_10_ odds ratio (OR) of 1 or more is indicated by the yellow color. (**C**) Disease characterizing signatures of LAM were constructed by comparing LAM1_cluster16_ with the WT cluster 9 and 12. Functional enrichment of top 200 most significantly (FDR < 0.05) up-regulated genes was carried out in terms of KEGG/MSigDB (Hallmark)/GO (Biological processes) categories. Selected functional classes based on the cutoff of FDR adjusted *p*-values < 0.1 are represented by different edge colors and size of the node is proportional to negative logarithm of FDR adjusted *p*-value.

Disease characterizing signature of LAM1 was constructed by comparing LAM1_cluster16_ with the transcriptionally analogous WT clusters. The analysis of overlaps between the LAM1_cluster16_ CAS and CASes of all WT clusters identified cells in WT clusters 9 and 12 (Figure 2A,B) as being the most similar to the LAM cells in LAM1_cluster16_. Single-cell disease characterizing signature (DCS) of LAM was then constructed by differential gene expression analysis between cells in LAM1_cluster16_ and cells in WT clusters 9 and 12. For comparison, we also constructed pseudo-bulk signature of LAM1 by differential expression between all LAM1 cells and all WT cells (Methods). This signature mimics the signature that would be obtained by the bulk RNA-seq analysis.

The pathway analysis of the LAM single-cell DCS against GO [18], KEGG [19], and MSigDB (Hallmark) [20] gene sets with clusterProfiler [21] implicated MTORC1 signaling hallmark gene sets as being enriched in the DCS (Figure 2C), along with gene sets and pathways associated with cell proliferation, invasion, and metastasis. Although most of these signaling pathways are known features of LAM, identifying their activity within the LAM cell populations based on a transcriptional signature is not a trivial task. The analysis of the pseudo-bulk LAM signature does not reveal increased MTOR signaling (Supplementary Figure S4A), demonstrating the critical increase in precision of our DCS in comparison to a typical signature constructed from bulk tissue profiling.

### 2.5. Connectivity Analysis

**Figure 3:**
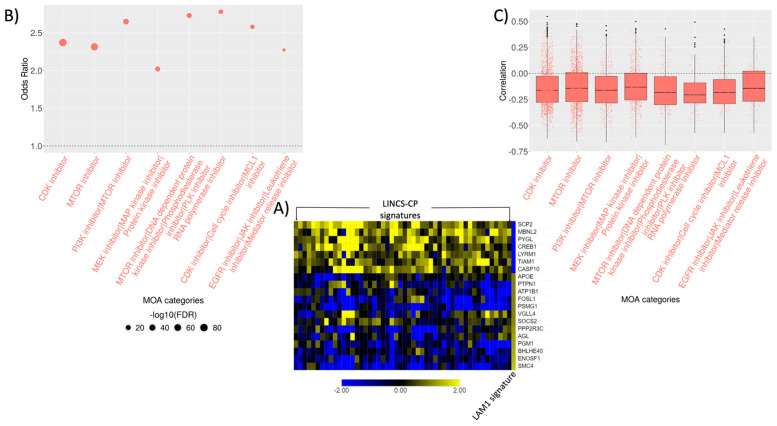
Connectivity analysis of naïve LAM signature. (**A**) Top 250 most up/down regulated genes from LAM characterizing signature were selected and matched with 978 LINCS-L1000 landmark genes. Pearson’s correlation were computed between each of the LINCS-CP and LAM signature. Top 50 LINCS-CP signatures most negatively correlated with the LAM signature (columns) with the corresponding matched genes (rows) are presented via heatmap. (**B**) Odds ratios of the top most enriched MOA categories are shown via dot plot where the size of the dots represents the significance of the MOA categories with a bigger dot indicating lower FDR adjusted *p*-value. MOA categories were selected based on odds ratio > 2, –log10(FDR) > 7, and at least 100 signatures in any MOA category. (**C**) Distribution of the overall signature correlations associated with each of the MOA categories is demonstrated via box-and-whisker plots. Each dot represents a LINCS-CP signature and negative correlations indicate the potential of the drug mechanisms to revert the LAM signature.

We developed methods for CMaP analysis of DCS’s with the aim of identifying the Mechanism of Action (MOA) of bioactive compounds capable of reversing the LAM. The single-cell DCS is first correlated with correlated with 143,374 LINCS signatures in response of 15,349 chemical perturbagens (CP) (Figure 3A). The enrichment of signature with high negative correlations among CPs with a specific MOA was then assessed using small-sample bias corrected logistic regression (technical details in Methods).

In the CMaP analysis of LAM1 DCS, most enriched MOA categories included MTOR inhibitors, dual inhibition of PI3K/MTOR, and CDK inhibitors (Figure 3B,C, and Supplementary Table S3). Given the known etiology of LAM, and the use of the sirolimus MTOR inhibitor in the treatment of LAM, ability of MTOR inhibitors to reverse the LAM was expected and also in line with the functional analysis results from the previous section. However, the same connectivity analysis repeated on the pseudo-bulk LAM signature failed to identify MTOR inhibitors as putative therapeutics (Supplementary Table S5). This demonstrates the importance of carefully constructing single-cell DCS for the successful connectivity analysis.

In terms of individual compounds, we found sirolimus, AZD-8055, OSI-027, and WYE-125132 showing consistently strong negative correlation across all the dosages with LAM1 DCS (Supplementary Figure S5A). Cyclin-dependent kinase inhibitors (CKI) [22,23] CGP-60474, PHA-793887, alvocidib (CDK1/2 inhibitors), and palbociclib (CDK4/6 inhibitor) also showed strong negative correlation with LAM1 single-cell DCS across different concentrations and cell lines (Supplementary Figure S5A).

Another class of compounds implicated by the connectivity and functional enrichment analysis of LAM1 DCS were MEK/MAP kinase/protein kinase inhibitors. Estrogen-induced activation of MAPK signaling was associated with enhanced cell proliferation [24] and survival of LAM cells [25]. Estrogen increased the expression of oncogene c-MYC, which plays a critical role in cell cycle progression by suppressing p21^Cip1^ expression [26], in LAM cells (Figure 3C) and might have induced MAPK signal transduction pathways [24,27]. Moreover, inhibition of mTORC1 is known to activate MAPK signaling cascade [28]. The combination therapies of concurrent inhibition of mTORC1 and MAPK are currently being investigated [29].

### 2.6. Signature Construction and Connectivity Analysis of Sirolimus Treated LAM

**Figure 4:**
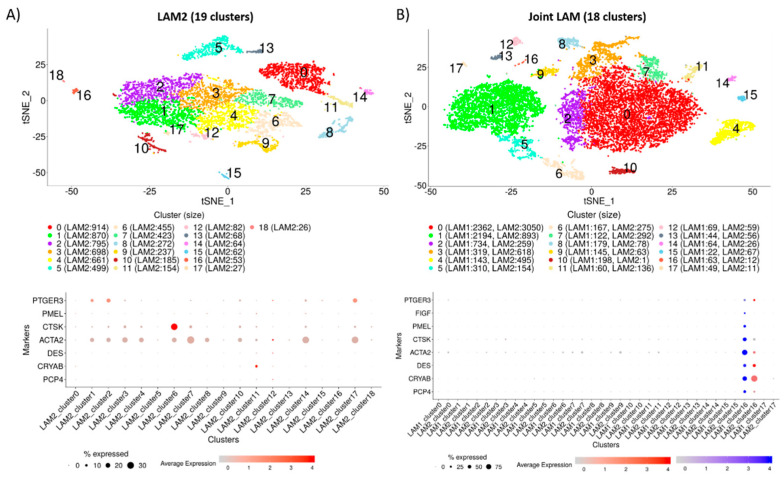
Cluster analysis of LAM2. (**A**) Unsupervised clustering of 6545 cells from LAM2 is represented in two-dimensional t-SNE plots (top panel) with perplexity 30. A total of 19 clusters were identified in each sample using Seurat’s graph-based clustering initialized with top principal components with largest variances. Expression of known LAM markers was used to identify the cluster of LAM cells (bottom panel), with the size of the dot representing the percentage of cells expressed and color is proportional to the average expression of the genes. (**B**) Integrated clustering of 13,789 cells from both LAM1 (7244 cells from LAM1) and LAM2 (6545 cells from LAM2) identified 18 clusters where each cluster consisted of both LAM1 and LAM2 cells (top panel). Seurat’s implementation of integrated clustering was used to identify common cell clusters between LAM1 and LAM2. Clusters are colored and labeled uniquely, and the number of cells in each cluster is noted inside the parenthesis in the legends. Six out of eight LAM markers show moderate to high expression in at least 25% cells in both LAM1 (63 cells) and LAM2 (12 cells) of cluster 16 (bottom panel).

Similar to naïve LAM, we repeated the analytical workflow for sirolimus-treated LAM sample (LAM2). The clustering algorithm identified 19 clusters in LAM2 (Figure 4A), and we used LAM marker genes to identify LAM cells in LAM2. However, unlike LAM1, the expression of LAM markers was not localized in any particular cluster, and cells expression were dispersed in all clusters making it impossible to identify a single LAM cluster (Supplementary Table S2). We hypothesized that the frequency of the LAM cells in LAM2 sample may be too low for them to be identified in the unsupervised fashion. As an alternative strategy, we combined LAM1 and LAM2 cells and re-clustered them.

A total of 13,789 cells from LAM1 and LAM2 were combined using Seurat’s [13] implementation of multiple dataset integration and 18 clusters were detected (Figure 4B). Briefly, the data were normalized and variable genes were identified separately for the two datasets. The “anchoring” pairs of cells in both datasets were identified by joint k-nearest neighbor analysis based on the cannonical correlation, and k = 5 and batch normalization was performed as described in the Seurat paper. Majority of the markers were highly expressed in both LAM1 and LAM2 part of cluster 16, which was further supported by the enrichment of LAM markers in the joint cluster (Supplementary Table S2). All the 57 cells from LAM1_cluster16_ were also present in the joint cluster 16. The 12 LAM2 cells in the joint cluster 16 were assumed to represent LAM cells in the LAM2 samples and were denoted as LAM2_joint-cluster16_. Please note that the fact that LAM cells clusters, the LAM1 sample (Figure 1), and combined clustering (Figure 4B) were both labeled as cluster 16 was purely coincidental.

**Figure 5:**
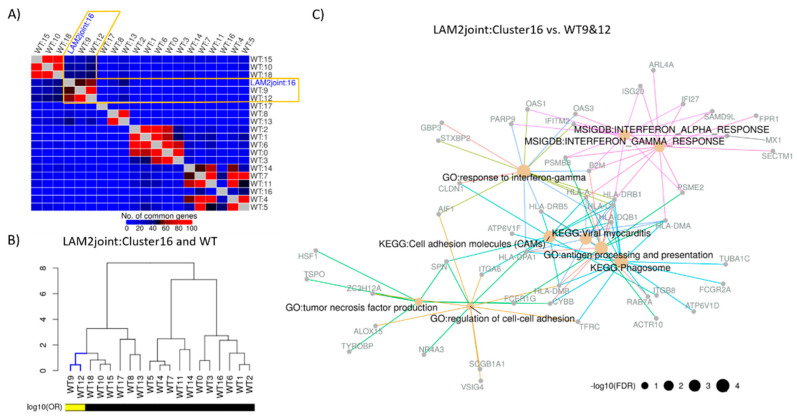
Construction and functional enrichment of disease characterizing signature from LAM2. (**A**) WT clusters were matched with the LAM2_joint-cluster16_ cluster in terms of top 200 most significantly (FDR < 0.05) up-regulated genes from each of the CAS. Unsupervised hierarchical clustering revealed sub-clusters of LAM and WT clusters, where LAM2_joint-cluster16_ was clustered with WT clusters 9 and 12. (**B**) Significance of the overlaps between LAM and WT cell clusters based on the significantly (FDR < 0.05) up-regulated genes were assessed via Fisher’s exact test. Cluster similarities were measured using log10 odds ratio and hierarchical clustering of LAM1_cluster16_ vs. WT is visualized via dendrograms. Log_10_ odds ratio (OR) of 1 or more is indicated by the yellow color. (**C**) Disease characterizing signatures of LAM were constructed by comparing LAM2_joint-cluster16_ with the WT cluster 9 and 12. Functional enrichment of top 200 most significantly (FDR < 0.05) up-regulated genes was carried out in terms of KEGG/MSigDB (Hallmark)/GO (Biological processes) categories. Selected functional classes based on the cutoff of FDR adjusted *p*-values < 0.05 and odds ratio > 2 are represented by different edge colors, and size of the node is proportional to negative logarithm of FDR adjusted *p*-value.

Cluster annotating signatures (significant genes listed in the Supplemental Tables) of the joint clusters showed similar cell and tissue types as in LAM1 analysis (Supplementary Figure S6). Cluster annotating signatures were further used to find the WT clusters akin to LAM2_joint-cluster16_. Similar to LAM1_cluster16_, WT cluster 9 and 12 had maximum number of overlapping genes with LAM2_joint-cluster16_ (Figure 5A,B). The single-cell DCS of LAM2 cells was constructed by differential gene expression analysis between cells in LAM2_joint-cluster16_ and the WT clusters 9 and 12. The pathway analysis of the LAM2 DCS identified pathways associated with the regulation of cell–cell adhesion, response to interferongamma, and tumor necrosis factor, but not MTOR signaling (Figure 5C). The list of all enriched pathways is provided in the Supplemental Tables.

**Figure 6:**
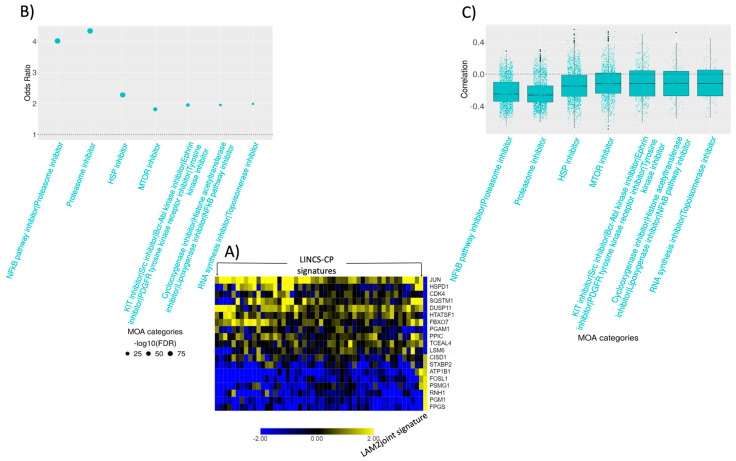
Connectivity analysis of sirolimus-treated LAM signature. (**A**) Top 250 most up/down regulated genes from LAM2 DCS signature was selected and matched with 978 LINCS-L1000 landmark genes. Pearson’s correlation was computed between each of the LINCS-CP and LAM signature. Top 50 LINCS-CP signatures most negatively correlated with the LAM signature (columns) and the corresponding matched genes (rows) are presented via heatmap. (**B**) Odds ratios of the most enriched MOA categories are shown via dot plot where the size of the dots represents the significance of the MOA categories with a bigger dot indicating lower FDR adjusted *p*-value. MOA categories were selected based on odds ratio > 1.75, −log10(FDR) > 4, and at least 150 signatures in any MOA category. (**C**) Distribution of the overall signature correlations associated with each of the MOA categories are demonstrated via box-and-whisker plots. Each dot represents a LINCS-CP signature, and negative correlations indicate the potential of the drug mechanisms to revert the LAM signature.

Connectivity analysis of LAM2 DCS (Figure 6A) revealed several MOA categories, including single-agent proteasome inhibitors, dual inhibition of NF-κB pathway/proteasome inhibitors, and HSP inhibitors. (Figure 6B, Supplementary Table S4). Mutation of TSC2 and its leading activation of MTORC1 upregulates the proteasome [30], which may facilitate estrogen-enhanced survival of tumor cells [31,32]. MTOR also activates NF-κB [33], a major regulator of cell survival, pro-inflammatory cytokines such as TNF-α, and cell adhesion molecules which may allow LAM cells to survive [34,35]. We also found response to interferon gamma and cell adhesion molecules in the functional enrichment of LAM2 DCS (Figure 6C), which might activate NF-κB and support the anti-apoptotic behavior of the LAM cells. Proteasome inhibitor, which inhibits NF-κB activation, has been found to reduce estrogen mediated survival of TSC2-null cells in LAM [32] and was one of the top hits in our connectivity analysis with LAM2 DCS. Signatures of tyrosine kinase and cyclooxygenase inhibitor drugs were also implicated (Figure 6B,C). Interestingly, several drugs related to this MOA, such as multi-kinase inhibitor imatinib, Src inhibitor Saracatinib, and Cyclooxygenase inhibitor Celecoxib, are being currently tested in clinical trials as LAM therapeutics, confirming the relevance of the connectivity analysis results. We also found MTOR inhibitors to be one of the most enriched MOA categories although with relatively low strength of association (odds ratios) (Figure 6B).

## 3. Discussion

The connectivity analysis leveraging large databases of transcriptional perturbation signatures such as LINCS-L1000 along with the open accessibility to processed transcriptomics data [36,37] and signatures [38,39] enables in silico discovery of novel therapeutics. However, disease-related biological processes and resulting transcriptional dysregulation are not uniform across all cell types within the diseased tissues. The differences in expression profiles between cells of different types usually dwarf the differences between diseased and non-diseased cells of the same type. Consequently, the cell-averaging in the traditional bulk assays can produce disease transcriptional signatures of no relevance for finding putative therapeutics via connectivity analysis. This has been clearly demonstrated in our analysis of LAM data. Our methodology for constructing and CMaP analysis of scRNA-seq signatures effectively circumvented this fundamental limitation.

The functional and CMaP analysis of LAM scRNA-seq signature firmly establishes the dysregulation of mTORC1 signaling as the primary target for therapeutic intervention and recapitulates known disease mechanism of LAM. At the same time, the analysis of corresponding pseudo-bulk signatures completely fails to establish the connection. To the best of our knowledge, this is the first analysis that describes and clearly demonstrates the importance of single-cell transcriptional signature based CMaP analysis. It is quite possible that a bulk analysis of more samples with different disease stages would have also identified the signature of mTOR signaling. Furthermore, our results are based on relatively few cells in a single sample of naïve LAM lung tissue, and analyses of additional samples will be necessary to establish the robustness of the results across different patients. However, the results still illustrate the power of scRNA-seq in constructing cell-type and patient-specific signatures and using them to search for promising therapies via CMaP analysis. Each of the steps in our analysis can be accomplished using a wide range of statistical methods, and breath-taking pace of methods development makes it difficult to choose the optimal methods [40,41]. For example, for the critical step of identifying the very small clusters of LAM cells we used graph-based Louvain-Jaccard clustering algorithm [42,43]. The fact that we were able to detect the LAM cell populations validates this choice. At the same time, using a different cluster analysis strategy and the parameters that determine the number of clusters [44,45], or even an ensemble of clustering results using different methodologies [46], may yield better results in another context. Similar considerations can lead to different choice of methodology for differential gene expression analysis [47], connectivity metrics, and MOA enrichment analysis of negatively correlated perturbation signatures. The rigorous benchmarking of different choices at each step would be difficult, and it is beyond the scope of this paper. We demonstrate that our methodology is conceptually sound and that choices we made are reasonable because they lead to positive results in a context of a very difficult problem where standard CMaP analysis methodologies would fail.

The analysis of LAM data here serves as proof of concept for general approach for pleiotropic effect of dysregulation of the mTOR signaling pathway in various human disorders [48,49,50] and aging itself [48,51,52]. mTOR inhibitors are currently the only pharmacological treatment shown to extend lifespan in model organisms [53,54,55], and mTOR signaling has been directly implicated in age-associated disorders such as Alzheimer’s disease [56]. Numerous inhibitors of mTOR signaling have been developed, and new drugs that modulate activity of mTOR signaling are under development [57]. Our results illustrate how CMaP analysis of disease scRNA-seq data can accurately detect mTOR dysregulation and predict mTOR signaling inhibitors as effective drugs when the classical CMaP of bulk tissues fails. The scRNA-seq data used in our analysis was previously described and analyzed by Guo et al. [12], and our pathway analysis results of naïve LAM signatures are consistent with results presented in that paper. Unlike Guo et al., we were also able to identify a small set of cells expressing known LAM markers in the sirolimus treated LAM sample. However, the most important contribution of our study is the CMaP of the LAM signatures.

In addition to mTORC1 inhibitors, our analysis also identified additional classes of drugs, as well as specific drugs, capable of reverting the LAM signature, such as antiproliferative CDK inhibitors, and MEK/MAPK inhibitors, which might induce cytotoxicity against the LAM cells. In this computational study, we make no attempts to experimentally validate any of these predictions and they remain speculative, although some of already been established and studies are under way to test effectiveness.

## 4. Materials and Methods

### 4.1. Single-Cell RNA-Seq and LINCS-L1000 Data

Single-cell RNA-seq (scRNA-seq) was performed on dissociated lung tissue samples that were collected from three different sources including an untreated LAM patient (LAM1); patient treated with sirolimus (LAM2); and a brain-dead, beating-heart, organ donor control patient (WT). Both LAM patients were undergoing lung transplantation. Single-cell suspensions of the two explanted LAM lungs and the normal lung were subjected to 10× Chromium scRNA-seq. CellRanger pipeline was used for read alignment and quantification. Raw gene counts data used in this analysis have been previously described and submitted to GEO [12] (GSE135851). LAM1 data correspond to the sample GSM4035465, LAM2 data correspond to sample GSM4035466, and WT sample corresponds to sample GSM4035472.

For connectivity analysis, we utilized LINCS-L1000 database, which is comprised of an extensive library of over a million gene expression profiles [11]. L1000 assay, a low-cost high-throughput technology developed by the Broad Institute, measures the expression of 978 landmark genes. The gene expression profiles were generated in response to a wide range of perturbing agents, including ~20,000 small molecule compounds in more than 100 human cell lines and cell types for a total of 473,647 signatures [10]. We considered 143,374 chemical perturbation signatures available via iLINCS [38] that were constructed by merging level-4 L1000 signature replicates into level-5 moderated Z-scores and only the reproducible signatures were retained.

### 4.2. Single-Cell RNA-Seq Data Pre-Processing and Clustering

For scRNA-seq data, we filtered low-quality cells that were expressed (unique molecular identifies (UMI) > 0) in less than 500 genes and had more than 10% mitochondrial UMI counts. Initial data pre-processing, normalization, and clustering were performed using Seurat3 [13] for LAM1, LAM2, and WT samples individually. Data were normalized by the global-scaling normalization method (“LogNormalize”), and top 2000 genes with highest standardized variance (method = “vst”) were selected for principal component (PC) analysis. For clustering, shared nearest-neighbor (SNN) graph was constructed with top 30 PCs with highest variances and Louvain algorithm for community detection [42] and resolution parameter of 0.8 was used for clustering of cells within each sample. For integrated clustering of LAM1 and LAM2, both samples were merged using “IntegrateData” based on the anchors from “FindIntegrationAnchors” object with default parameters in Seurat3. Resolution parameter was set to 0.4 for cell clustering in the integrated LAM.

### 4.3. Construction of Cluster Annotating and Disease Characterizing Signatures

We employed a two-step strategy to annotate cell clusters and construct disease characterizing signature. In step 1, pairwise differential expression (DE) of each cluster was computed using MAST [47] Bioconductor package, which generated $n_{t}-1$ DE for each cluster (Supplementary Figure S1A), where $n_{t}$ is the number of clusters in sample *t*. For each pairwise comparison, we calculated π-score [58] by multiplying log2 fold change (LFC) and negative logarithm of *p-*values (corrected for multiple testing using Benjamini-Hochberg (BH) method [59]). This can be written as $\pi_{irc}=\varphi_{irc}\cdot \left(-log_{10}P_{irc}\right)$, where $\varphi_{irc,}$ and $P_{irc}$ are LFC and *p*-values for $i^{th}$ gene, $r^{th}$ comparison, and $c^{th}$ cluster, respectively. A positive π score indicates an up-regulation of a gene, whereas a negative score means down-regulation. A one-sided one sample Student’s *t*-test was carried out to combine the $n_{t}-1$ DEs into a cluster specific signature under the following hypotheses:

$H_{0}:\mu_{ic}^{\pi }=\mu_{0}\;vs.H_{1}:\mu_{ic}^{\pi }>\mu_{0}$, where $\mu_{ic}^{\pi }$ is the mean π score for gene i and cluster c.

The null value was considered as 2 based on the cutoff of a gene being called differentially upregulated with pre-specified LFC of 1 and *p-*value of 0.01. *p*-values from *t-*test were further corrected for multiple testing using Benjamini–Hochberg method [59]. Top 200 most significantly (FDR < 0.05) up-regulated genes were considered for cell-type/tissue enrichment via CLEAN [60]. The cluster of disease-critical LAM cells was identified as the one most enriched for 8 LAM marker genes.

In step 2, LAM-specific cell cluster (LAM_cluster16_) was matched with WT clusters in terms of top 200 differentially upregulated (DU) genes (Supplementary Figure S1A). Similarities between LAM and WT clusters based on the number of overlapping genes were determined using complete linkage-based hierarchical clustering with Euclidean distance measure. Significance of the overlaps among LAM and WT clusters was assessed via Fisher’s exact test. Finally, disease characterizing signature of both LAM1 and LAM2 was constructed by comparing LAM1 cells and LAM2 cells from LAM_cluster16_ with the matched WT clusters separately. Pseudo-bulk signatures for LAM1 and LAM2 were constructed by comparing all the LAM1 cells with WT cells and LAM2 cells with the WT cells respectively using MAST [47] Bioconductor package.

### 4.4. Connectivity Analysis

LINCS-L1000 chemical perturbational (CP) signatures were considered for connectivity analysis. We selected 250 most significantly (FDR < 0.05) differentially expressed (125 up-regulated and 125 down-regulated) genes from the LAM characterizing signature and matched them with the 978 L1000 landmark genes. Let $Q_{i}$ be the LAM signature and $L_{ij}$ be the LINCS-CP signatures, where *i* is the set of matched genes and *j* is the set of LINCS CP signatures. Pearson correlation $Cor_{j}\left(Q,L_{j}\right)$ was computed between LAM and each of the LINCS CP signatures (Supplementary Figure S1B) to assess the strength of relationship between the signatures. Negative correlation *p*-values were calculated for each signature correlation. A total of 86,538 LINCS CP signatures associated with 1005 unique mechanism of action (MOA) categories corresponding to the small molecules/drugs were considered for further MOA enrichment.

Let M be a binary variable, where

(1) $$m_{k}=\{\begin{array}{c} 1,\;for\;the\;k^{th}\;MOA\;category\\ 0,\;for\;all\;other\;categories \end{array}$$

Here, k=1,2,……,1005. Inspired by the LRpath method [61], we then fitted a small sample bias corrected binary logistic regression model [62] for  M: $logit\left(\mathrm{Pr}\left(M_{k}=1\right)\right)=X_{k}^{T}\beta$, where negative logarithm of down-regulated *p*-values of correlation between LAM and LINCS-CP signatures is the predictor variable (Supplementary Figure S1B). β>0 indicates that the signatures of the drugs for a specific MOA are “connected” with the disease signatures.

## Data Availability

All data used has been previously described and is publicly available.

## Supplementary Materials

The following are available online at https://www.mdpi.com/article/10.3390/ijms22094371/s1. Supplemental file 1, Supplemental Results; Supplemental_Excel_Spreadsheets.

## References

[1] McCormack F.X., Inoue Y., Moss J., Singer L.G., Strange C., Nakata K., Barker A.F., Chapman J.T., Brantly M.L., Stocks J.M. (2011). Efficacy and safety of sirolimus in lymphangioleiomyomatosis. N. Engl. J. Med..

[2] Bissler J.J., McCormack F.X., Young L.R., Elwing J.M., Chuck G., Leonard J.M., Schmithorst V.J., Laor T., Brody A.S., Bean J. (2008). Sirolimus for angiomyolipoma in tuberous sclerosis complex or lymphangioleiomyomatosis. N. Engl. J. Med..

[3] Taveira-DaSilva A.M., Moss J. (2012). Optimizing treatments for lymphangioleiomyomatosis. Expert Rev. Respir. Med..

[4] Sirota M., Dudley J.T., Kim J., Chiang A.P., Morgan A.A., Sweet-Cordero A., Sage J., Butte A.J. (2011). Discovery and preclinical validation of drug indications using compendia of public gene expression data. Sci. Transl. Med..

[5] Pushpakom S., Iorio F., Eyers P.A., Escott K.J., Hopper S., Wells A., Doig A., Guilliams T., Latimer J., McNamee C. (2019). Drug Repurposing: Progress, challenges and recommendations. Nat. Rev. Drug Discov..

[6] Ashburn T.T., Thor K.B. (2004). Drug repositioning: Identifying and developing new uses for existing drugs. Nat. Rev. Drug Discov..

[7] Lamb J., Crawford E.D., Peck D., Modell J.W., Blat I.C., Wrobel M.J., Lerner J., Brunet J.-P., Subramanian A., Ross K.N. (2006). The connectivity map: Using gene-expression signatures to connect small molecules, genes, and disease. Science.

[8] Claerhout S., Lim J.Y., Choi W., Park Y.-Y., Kim K., Kim S.-B., Lee J.-S., Mills G.B., Cho J.Y. (2011). Gene expression signature analysis identifies vorinostat as a candidate therapy for gastric cancer. PLoS ONE.

[9] Dudley J.T., Sirota M., Shenoy M., Pai R.K., Roedder S., Chiang A.P., Morgan A.A., Sarwal M.M., Pasricha P.J., Butte A.J. (2011). Computational repositioning of the anticonvulsant topiramate for inflammatory bowel disease. Sci. Transl. Med..

[10] Subramanian A., Narayan R., Corsello S.M., Peck D.D., Natoli T.E., Lu X., Gould J., Davis J.F., Tubelli A.A., Asiedu J.K. (2017). A next generation connectivity map: L1000 platform and the first 1,000,000 profiles. Cell.

[11] Keenan A.B., Jenkins S.L., Jagodnik K.M., Koplev S., He E., Torre D., Wang Z., Dohlman A.B., Silverstein M.C., Lachmann A. (2018). The library of integrated network-based cellular signatures NIH program: System-level cataloging of human cells response to perturbations. Cell Syst..

[12] Guo M., Yu J.J., Perl A.K., Wikenheiser-Brokamp K.A., Riccetti M., Zhang E.Y., Sudha P., Adam M., Potter A., Kopras E.J. (2020). Single cell transcriptomic analysis identifies a unique pulmonary lymphangioleiomyomatosis cell. Am. J. Respir. Crit. Care Med..

[13] Stuart T., Butler A., Hoffman P., Hafemeister C., Papalexi E., Mauck III W.M., Hao Y., Stoeckius M., Smibert P., Satija R. (2019). Comprehensive integration of single-cell data. Cell.

[14] Han X., Zhou Z., Fei L., Sun H., Wang R., Chen Y., Chen H., Wang J., Tang H., Ge W. (2020). Construction of a human cell landscape at single-cell level. Nature.

[15] Zhang X., Lan Y., Xu J., Quan F., Zhao E., Deng C., Luo T., Xu L., Liao G., Yan M. (2019). CellMarker: A manually curated resource of cell markers in human and mouse. Nucleic Acids Res..

[16] Franzén O., Gan L.-M., Björkegren J.L.M. (2019). PanglaoDB: A web server for exploration of mouse and human single-cell RNA sequencing data. Database.

[17] Su A.I., Wiltshire T., Batalov S., Lapp H., Ching K.A., Block D., Zhang J., Soden R., Hayakawa M., Kreiman G. (2004). A Gene Atlas of the Mouse and Human Protein-Encoding Transcriptomes. Proc. Natl. Acad. Sci. USA.

[18] Ashburner M., Ball C.A., Blake J.A., Botstein D., Butler H., Cherry J.M., Davis A.P., Dolinski K., Dwight S.S., Eppig J.T. (2000). Gene ontology: Tool for the unification of biology. Nat. Genet..

[19] Kanehisa M., Goto S. (2000). KEGG: Kyoto encyclopedia of genes and genomes. Nucleic Acids Res..

[20] Liberzon A., Birger C., Thorvaldsdóttir H., Ghandi M., Mesirov J.P., Tamayo P. (2015). The molecular signatures database hallmark gene set collection. Cell Syst..

[21] Yu G., Wang L.-G., Han Y., He Q.-Y. (2012). ClusterProfiler: An R package for comparing biological themes among gene clusters. Omi. A J. Integr. Biol..

[22] Besson A., Dowdy S.F., Roberts J.M. (2008). CDK inhibitors: Cell cycle regulators and beyond. Dev. Cell.

[23] Malumbres M., Barbacid M. (2009). Cell cycle, CDKs and cancer: A changing paradigm. Nat. Rev. Cancer.

[24] Yu J., Astrinidis A., Howard S., Henske E.P. (2004). Estradiol and tamoxifen stimulate LAM-associated angiomyolipoma cell growth and activate both genomic and nongenomic signaling pathways. Am. J. Physiol. Cell. Mol. Physiol..

[25] Jane J.Y., Robb V.A., Morrison T.A., Ariazi E.A., Karbowniczek M., Astrinidis A., Wang C., Hernandez-Cuebas L., Seeholzer L.F., Nicolas E. (2009). Estrogen promotes the survival and pulmonary metastasis of tuberin-null cells. Proc. Natl. Acad. Sci. USA.

[26] Seoane J., Le H.-V., Massagué J. (2002). Myc suppression of the P21 Cip1 cdk inhibitor influences the outcome of the P53 response to DNA damage. Nature.

[27] Gramling M.W., Eischen C.M. (2012). Suppression of ras/mapk pathway signaling inhibits myc-induced lymphomagenesis. Cell Death Differ..

[28] Carracedo A., Ma L., Teruya-Feldstein J., Rojo F., Salmena L., Alimonti A., Egia A., Sasaki A.T., Thomas G., Kozma S.C. (2008). Inhibition of MTORC1 leads to MAPK pathway activation through a PI3K-dependent feedback loop in human cancer. J. Clin. Investig..

[29] Mi R., Ma J., Zhang D., Li L., Zhang H. (2009). Efficacy of combined inhibition of MTOR and ERK/MAPK pathways in treating a tuberous sclerosis complex cell model. J. Genet. Genomics.

[30] Zhang Y., Nicholatos J., Dreier J.R., Ricoult S.J.H., Widenmaier S.B., Hotamisligil G.S., Kwiatkowski D.J., Manning B.D. (2014). Coordinated regulation of protein synthesis and degradation by MTORC1. Nature.

[31] Johnson C.E., Dunlop E.A., Seifan S., McCann H.D., Hay T., Parfitt G.J., Jones A.T., Giles P.J., Shen M.H., Sampson J.R. (2018). Loss of tuberous sclerosis complex 2 sensitizes tumors to nelfinavir-bortezomib therapy to intensify endoplasmic reticulum stress-induced cell death. Oncogene.

[32] Li C., Li N., Liu X., Zhang E.Y., Sun Y., Masuda K., Li J., Sun J., Morrison T., Li X. (2016). Proapoptotic protein bim attenuates estrogen-enhanced survival in lymphangioleiomyomatosis. JCI Insight.

[33] Karin M. (2006). Nuclear factor-ΚB in cancer development and progression. Nature.

[34] Henske E.P., McCormack F.X. (2012). Lymphangioleiomyomatosis—A wolf in sheep’s clothing. J. Clin. Investig..

[35] Ghosh S., Tergaonkar V., Rothlin C.V., Correa R.G., Bottero V., Bist P., Verma I.M., Hunter T. (2006). Essential role of tuberous sclerosis genes TSC1 and TSC2 in NF-ΚB activation and cell survival. Cancer Cell.

[36] Al Mahi N., Najafabadi M.F., Pilarczyk M., Kouril M., Medvedovic M. (2019). GREIN: An interactive web platform for re-analyzing GEO RNA-Seq data. Sci. Rep..

[37] Athar A., Füllgrabe A., George N., Iqbal H., Huerta L., Ali A., Snow C., Fonseca N.A., Petryszak R., Papatheodorou I. (2019). ArrayExpress update–from bulk to single-cell expression data. Nucleic Acids Res..

[38] Pilarczyk M., Najafabadi M.F., Kouril M., Vasiliauskas J., Niu W., Shamsaei B., Mahi N., Zhang L., Clark N., Ren Y. (2019). Connecting omics signatures of diseases, drugs, and mechanisms of actions with ILINCS. bioRxiv.

[39] Wang Z., Monteiro C.D., Jagodnik K.M., Fernandez N.F., Gundersen G.W., Rouillard A.D., Jenkins S.L., Feldmann A.S., Hu K.S., McDermott M.G. (2016). Extraction and analysis of signatures from the gene expression omnibus by the crowd. Nat. Commun..

[40] Ding J., Adiconis X., Simmons S.K., Kowalczyk M.S., Hession C.C., Marjanovic N.D., Hughes T.K., Wadsworth M.H., Burks T., Nguyen L.T. (2020). Systematic comparison of single-cell and single-nucleus RNA-sequencing methods. Nat. Biotechnol..

[41] Vieth B., Parekh S., Ziegenhain C., Enard W., Hellmann I. (2019). A systematic evaluation of single cell RNA-seq analysis pipelines. Nat. Commun..

[42] Blondel V.D., Guillaume J.-L., Lambiotte R., Lefebvre E. (2008). Fast unfolding of communities in large networks. J. Stat. Mech. Theory Exp..

[43] Levine J.H., Simonds E.F., Bendall S.C., Davis K.L., El-ad D.A., Tadmor M.D., Litvin O., Fienberg H.G., Jager A., Zunder E.R. (2015). Data-driven phenotypic dissection of AML reveals progenitor-like cells that correlate with prognosis. Cell.

[44] Bahlo M., Tian L., Lönnstedt I., Ng M., Hicks S. (2018). Comparison of clustering tools in R for medium-sized 10× genomics single-cell RNA-sequencing. F1000 Res..

[45] Duò A., Robinson M.D., Soneson C. (2018). A Systematic Performance Evaluation of Clustering Methods for Single-Cell RNA-Seq Data. F1000 Res..

[46] Yang Y., Huh R., Culpepper H.W., Lin Y., Love M.I., Li Y. (2019). SAFE-clustering: Single-cell aggregated (from ensemble) clustering for single-cell RNA-seq data. Bioinformatics.

[47] Finak G., McDavid A., Yajima M., Deng J., Gersuk V., Shalek A.K., Slichter C.K., Miller H.W., McElrath M.J., Prlic M. (2015). MAST: A Flexible statistical framework for assessing transcriptional changes and characterizing heterogeneity in single-cell RNA sequencing data. Genome Biol..

[48] Saxton R.A., Sabatini D.M. (2017). MTOR signaling in growth, metabolism, and disease. Cell.

[49] Carsillo T., Astrinidis A., Henske E.P. (2000). Mutations in the tuberous sclerosis complex gene TSC2 are a cause of sporadic pulmonary lymphangioleiomyomatosis. Proc. Natl. Acad. Sci. USA.

[50] McCormack F.X., Travis W.D., Colby T.V., Henske E.P., Moss J. (2012). Lymphangioleiomyomatosis: Calling it what it is: A low-grade, destructive, metastasizing neoplasm. Am. J. Respir. Crit. Care Med..

[51] Stallone G., Infante B., Prisciandaro C., Grandaliano G. (2019). MTOR and Aging: An old fashioned dress. Int. J. Mol. Sci..

[52] Liu G.Y., Sabatini D.M. (2020). MTOR at the nexus of nutrition, growth, ageing and disease. Nat. Rev. Mol. Cell Biol..

[53] Robida-Stubbs S., Glover-Cutter K., Lamming D.W., Mizunuma M., Narasimhan S.D., Neumann-Haefelin E., Sabatini D.M., Blackwell T.K. (2012). TOR signaling and rapamycin influence longevity by regulating SKN-1/Nrf and DAF-16/FoxO. Cell Metab..

[54] Bjedov I., Toivonen J.M., Kerr F., Slack C., Jacobson J., Foley A., Partridge L. (2010). Mechanisms of life span extension by rapamycin in the fruit fly drosophila melanogaster. Cell Metab..

[55] Harrison D.E., Strong R., Sharp Z.D., Nelson J.F., Astle C.M., Flurkey K., Nadon N.L., Wilkinson J.E., Frenkel K., Carter C.S. (2009). Rapamycin fed late in life extends lifespan in genetically heterogeneous mice. Nature.

[56] Spilman P., Podlutskaya N., Hart M.J., Debnath J., Gorostiza O., Bredesen D., Richardson A., Strong R., Galvan V. (2010). Inhibition of MTOR by rapamycin abolishes cognitive deficits and reduces amyloid-β levels in a mouse model of alzheimer’s disease. PLoS ONE.

[57] Zheng Y., Jiang Y. (2015). MTOR Inhibitors at a glance. Mol. Cell. Pharmacol..

[58] Xiao Y., Hsiao T.-H., Suresh U., Chen H.-I.H., Wu X., Wolf S.E., Chen Y. (2014). A novel significance score for gene selection and ranking. Bioinformatics.

[59] Benjamini Y., Hochberg Y. (1995). Controlling the false discovery rate: A practical and powerful approach to multiple testing. J. R. Stat. Soc. Ser. B.

[60] Freudenberg J.M., Joshi V.K., Hu Z., Medvedovic M. (2009). CLEAN: CLustering enrichment ANalysis. BMC Bioinform..

[61] Sartor M.A., Leikauf G.D., Medvedovic M. (2009). LRpath: A logistic regression approach for identifying enriched biological groups in gene expression data. Bioinformatics.

[62] Kosmidis I., Pagui E.C.K., Sartori N. (2020). Mean and median bias reduction in generalized linear models. Stat. Comput..

